# Heterogeneity in Alzheimer’s Disease and Related Dementias

**DOI:** 10.20900/agmr20190010

**Published:** 2019-08-19

**Authors:** Rhoda Au

**Affiliations:** 1 Department of Neurology, Boston University School of Medicine, Boston, MA 02118, USA;; 2 Framingham Heart Study, National Heart, Lung, and Blood Institute, Boston, MA 01702, USA; 3 Department of Epidemiology, Boston University School of Public Health, Boston, MA 02118, USA; 4 Department of Anatomy & Neurobiology, Boston University School of Medicine, Boston, MA 02118, USA

Despite billions of dollars spent, there is still no effective pharmacotherapies for Alzheimer’s disease (AD). The problem plaguing AD research may well lie at the level of study design/data collection. Because we depend on a peer-reviewed system for funding, in order to get a fundable grant in AD, we all have to meet pre-conceived criteria for appropriate methodology, which has led us all to essentially propose similar study designs and collect essentially the same data. So not shockingly, we are all finding the same thing. And our evidence based approach for determining treatments is leading to the same path of failed clinical trial studies.

Now let’s consider the current rage of lifestyle risk factors as interventional strategy for reducing AD risk, an arena of research that Kristine Yaffe, Ph.D. at University of California, San Francisco, should be noted as having touted for decades. The modest success of the Finnish Geriatric Intervention Study to Prevent Cognitive Impairment and Disability (FINGER) study has led to an international network of FINGER studies cropping up across the world, with the U.S. doing its own version, the Protect Brain Health Through Lifestyle Intervention to Reduce Risk (POINTER) study [1]. These are largely fueled by our knowledge that cardiovascular risk factors are linked to accelerated brain aging/increased risk for dementia/AD. Why is that? Because of AD studies that in the U.S., have capitalized on longitudinal studies funded through the NHLBI, the most famous of which is the Framingham Heart Study (FHS) [2].

The heart-brain link derives from studies that were not originally designed as dementia/AD studies. Thus they are enriched with data that a typically designed AD study does not collect. We have also begun to understand dementia/AD as a life course disease. This is in part driven also by studies like FHS. Because unlike all the AD studies in which study inclusion criteria usually restricts the minimum age of the study sample to age 60 or 65 years and older, FHS, through its multi-generational design, has been able to provide evidence that mid-life health matters, and more recently early adult health matters because enrollment of their subjects span the entire adult lifespan.

These research results spur the idea that actually aging is not something that happens in late life, but is in fact a conception to death process and that to fully understand lifetime risk of dementia/AD you have to actually study the entire lifespan. When we start studying dementia/AD outside the boundaries of what is presumed to be known about the disease is when we will fully start to characterize it. And when we take this approach, instead of centering on the goal of AD precision medicine, this leads us to think about the entire continuum of brain aging. This, in turn, leads to a more comprehensive focus on precision brain health, where the goal is to optimize brain health wherever a person aligns on the normal to disease continuum. The shift in mindset to a precision brain health approach of finding the right solution for the right person at the right time replaces our current AD medicine focus of finding the right treatment for the right person at the right time.

In the special issue “Heterogeneity in AD and Related Dementias”, I welcome discussions tapping into other disease states to better understand their relationship to brain aging. I look forward to aggregating data that could help us embrace the full complexity of dementia/AD as the path to understanding it.
